# Influences of electrode density on intracranial seizure localisation: a single-blinded randomised crossover study

**DOI:** 10.1016/j.ebiom.2025.105606

**Published:** 2025-03-03

**Authors:** Ebenezer O. Chinedu-Eneh, Sharon Chiang, John P. Andrews, Ehsan Tadayon, Joline M. Fan, Paul A. Garcia, Ernesto Gonzalez-Giraldo, Manu Hegde, Patrick Hullett, Vikram R. Rao, Robert C. Knowlton, Edward F. Chang, Jonathan K. Kleen

**Affiliations:** a Department of Neurology, University of California San Francisco, San Francisco, CA, USA; b Weill Institute for Neurosciences, University of California San Francisco, San Francisco, CA, USA; c Department of Neurological Surgery, University of California San Francisco, San Francisco, CA, USA; d Department of Neurology, Tufts University, Boston, MA, 02116, USA

**Keywords:** Electrocorticography, Stereo-EEG, Depth, Grid, Epilepsy, Density, Intracranial, Subdural

## Abstract

**Background:**

Successful seizure onset zone (SOZ) localisation for epilepsy surgery often relies upon intracranial recordings. Accurate delineation requires anatomical detail yet influences of intracranial electrode density on clinical variables have not been systematically studied.

**Methods:**

In this experimental study we compared SOZ localisation between spontaneously captured seizures on higher-density depth and grid electrode arrays (4–5 mm inter-electrode spacing) vs. lower-density resampled versions of those same seizures (8–10 mm spacing). Since traditional review of channel traces would reveal density conditions, we instead projected seizure activity data as heatmaps on patient brain reconstructions and hid electrode locations. Using a single-blinded randomised crossover design, six attending-level epileptologists viewed these visualisations from ten patients under both higher-density and lower-density conditions (n = 120 observations) and digitally annotated SOZs.

**Findings:**

Inter-rater agreement between epileptologists on annotated margins was moderate (average Cohen's kappa: 0.47) and lower for the lower-density condition (p = 0.021, mixed effects model). Scorer confidence ratings did not differ between higher- and lower-density conditions (p = 0.410). The spatial extents of annotated SOZs for higher-density recordings were 25.4% larger on average (p = 0.011) and always closer to true SOZ extents in computer simulations, relative to lower-density.

**Interpretation:**

Epileptologists using higher-density depth and subdural intracranial EEG recordings had higher inter-rater agreement and identified larger extents of SOZs compared to lower-density recordings. While further studies assessing surgical outcomes in more patients are needed, these results suggest higher densities of electrodes on already-implanted hardware may reveal sub-centimetre extensions and clearer functional contiguity of the SOZ(s) for better appraisals of pathophysiological margins in epilepsy surgery.

**Funding:**

This work was supported by the 10.13039/100000002National Institutes of Health through 10.13039/100000065NINDS grant K23NS110920 and through a UCSF Weill Institute for Neurosciences Pilot Award.

**Research in context**

**Evidence before this study**

We reviewed existing literature (PubMed search) on intracranial electroencephalography (ICEEG) and electrode density regarding their role in epilepsy surgery, particularly focusing on seizure onset zone (SOZ) localisation. Our search, updated through February 1st 2024, included terms “(intracranial) AND (localisation) AND (surgery) AND (density).” Multiple studies evaluated higher-density EEG yet with scalp electrodes. One intracranial study assessed digital surface area alteration as opposed to subsampling, while another study used intracranial electrode subsampling comparisons yet for sLORETA source imaging on interictal discharges for a single patient. We have included in our references these and other studies that examine electrode densities in ICEEG for research-related goals (e.g. speech decoding). No study to our knowledge has evaluated intracranial seizure localisation using resampled electrode arrays, nor applied recent advances in seizure data visualisation to accomplish a blinded study design.

**Added value of this study**

This research adds value to existing evidence by providing a direct and unbiased comparison of how electrode density influences multiple clinically relevant variables for seizure localisation. Judgements of fully trained epileptologists applied to anatomical accuracy, subjective confidence, and inter-rater reliability were assessed using balanced and tightly controlled mixed effect statistical models. Our approach utilised a single-blinded randomised crossover design and quantitative clinical data visualisation techniques for new insights into spatial detail influences during SOZ delineation. Our findings suggest that HD ICEEG recordings offer better inter-rater reliability and more precise localisation of the SOZ, crucial factors for successful epilepsy surgery outcomes.

**Implications of all the available evidence**

The results of our study demonstrate clinical benefits for human health regarding the use of higher densities of electrode arrays in ICEEG for epilepsy surgery. This influence may be analogous to the importance of increased resolution in neuro-imaging over the past few decades (e.g. tumour margin estimation), despite being different modalities and contexts. In other words, by influencing judgement on tissue margins and the diagnostic agreement between clinicians, the spatial resolution of ICEEG as a functional diagnostic test holds strong clinical implications for epilepsy surgery. Specifically, increasing the sheer number of electrodes on already-implanted depth, strip, and grid arrays may lead to a better understanding of disease in terms of SOZ localisation, and by extension plausibly better surgical outcomes though prospective studies with larger patient numbers are needed. This study also highlights the need for further research into optimising ideal electrode densities in ICEEG recordings, particularly for stereo-EEG which has become the dominant ICEEG method. This work is timely considering parallel advances in higher density arrays and their increasing clinical promise.

## Introduction

Examining intracranial electroencephalography (ICEEG) recordings to characterise the seizure onset zone (SOZ) is crucial to epilepsy surgery. If there is no concern for eloquent function in the SOZ, the margins of tissue proposed for removal are defined by whether they are in the SOZ vs. those that are not. Much of this decision hinges upon the details of the ICEEG recordings, and the accuracy of this delineation–hence the removal of the SOZ–is key to successful epilepsy surgery.^1^^,^^2^

Delineating the SOZ and its margins is predicated on sufficient spatial sampling, meaning having enough electrodes in and around the SOZ.^3^^,^^4^ Traditional epilepsy surgery recordings have used subdural grid electrode arrays placed over the SOZ, and their conventional inter-electrode spacing is generally 10 mm which is assumed to be sufficient for sub-lobar SOZ delineation.^5^, ^6^, ^7^ Linear depth electrode arrays with inter-electrode spacing of 5–10 mm can be combined with subdural grid array recordings in order to simultaneously sample from deep brain structures.^7^ Most centres now use depth electrode arrays exclusively (several or even dozens) in the brain to localise the SOZ, referred to as stereo-EEG (SEEG).^8^^,^^9^ However, there is often substantial space between grid to depth, and depth to depth, leaving swaths of cortex unsampled. Unless sufficiently dense in the right regions, defining SOZ margins can be challenging considering the large areas of unsampled tissue.^7^

Despite these presumed concerns, it remains unknown whether a higher density of electrodes on already-implanted hardware components is more effective for determining the SOZ and its margins relative to a lower density. While more detail is often better (e.g. video screen resolution), higher electrode density in subdural or depth arrays would bring trade-offs of increased complexity in recordings (amplifiers with higher channel capacity, larger data storage and mobilisation needs) and in clinical interpretation (i.e., larger numbers of channels).

**Fig. 1:**
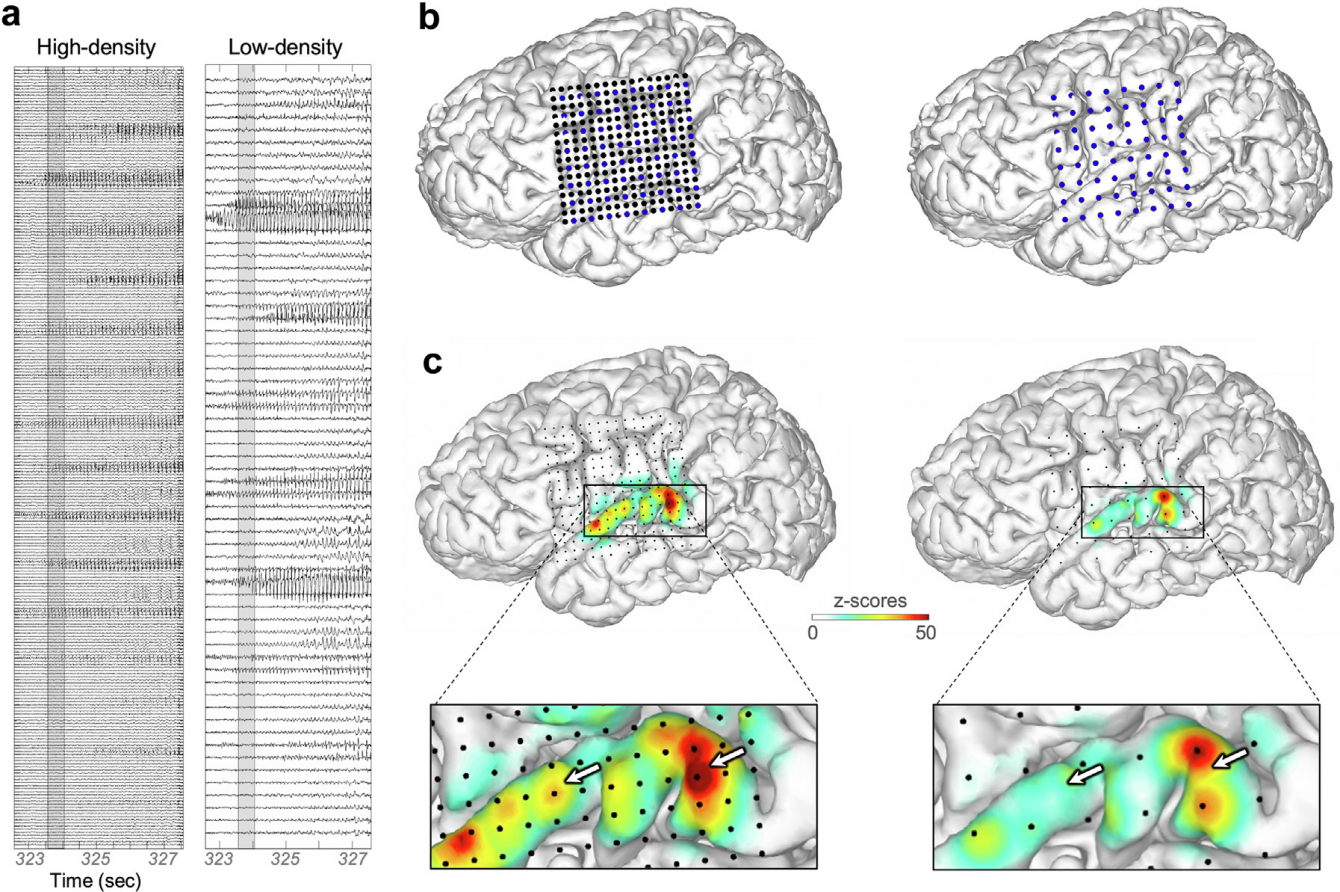
**HD and LD intracranial SOZ data visualisation. a.** Trace-based ICEEG recording within seconds of seizure onset using HD electrodes (left) and subsampled LD version used for clinical purposes (right). **b.** Reconstructed personalised brain of Patient 1 and the HD recording locations (dots, left panel). Blue dots indicate the subsampled version electrode contacts utilised to approximate LD density spacing of many clinical recordings, shown in the right panel. **c.** For each seizure, OPSCEA videos were rendered for both HD (left) and subsampled LD (right) versions. Electrode locations (small dots) and projection of seizure intensity (line-length transform)^11^ are shown during an example frame (corresponding grey windows in A) just after ictal onset to demonstrate the subtle differences in heatmap spatial detail. This includes adjacent HD sites (arrows, lower panels) that show similar or more activity in the HD version, but which are not available in the LD version, potentially affecting the interpretation of SOZ activity intensity, area, and contiguity.

The current study was driven by the unacceptable current rates of patients requiring repeat epilepsy surgery (particularly non-lesional cases)^10^ which may stem from subtotal estimation and resection of the SOZ, and observations that higher densities of electrodes show richer spatial detail of epileptiform activity (Fig. 1a).^12^^,^^13^ Whereas prior studies assessing density influences have largely focused on scalp EEG, magnetoencephalography (MEG), or interictal discharges,^14^^,^^15^ we hypothesise that there is a clinical benefit of ICEEG recordings at higher density (SEEG-depth and/or subdural arrays) compared to conventional recordings.

We used a randomised single-blinded crossover approach to directly compare higher-density (HD) electrode sampling vs. lower-density (LD) sampling in terms of anatomical accuracy, reviewer confidence, and other clinically relevant measures of SOZ delineation. Standard ICEEG review (trace-based) is not suited to answer this question since scorers would see obvious disparities in a number of electrodes (up to four times as many for higher-density grids), preventing blinding and thus risking bias. Therefore, we instead utilised a data visualisation technique projecting the level of epileptiform activity as a heatmap on patient-specific brain reconstructions (omni-planar and surface casting of epileptiform activity; OPSCEA).^11^ This approach has been recently validated to provide direct visualisation of onset and propagation patterns with clinical relevance^5^^,^^11^^,^^16^^,^^17^ and it enables direct comparison of blinded LD vs. HD conditions.

## Methods

### ICEEG recordings and patient selection

Our centre (UCSF Medical Center, Parnassus Campus, San Francisco, CA) has routinely implanted HD electrode grid arrays (in combination with strips and depth probes; termed “hybrid recordings”.^7^) in about half of patients undergoing ICEEG recordings since 2012, the rest being SEEG (depths only). The signal is split to enable intermittent (5–10 min) recordings from all HD electrode for research (Tucker Davis Technologies 512-channel amplifier) and 24/7 continuous clinical recordings from every other row and column of the grid (Fig. 1a). These “subsampled” or simulated LD recording versions serve to reduce the number of channels to within clinical amplifier capacities (256-channel Quantum Amplifier, Natus Medical Inc.). This maintains a roughly similar extent of anatomical coverage while increasing the spacing between electrodes similar to many clinical arrays (10 mm spacing^18^, ^19^, ^20^). All electrodes from depth (5 mm spacing) and strip electrodes (10 mm) are recorded on both research and clinical systems.

Occasionally, a spontaneous seizure occurs while a patient is actively participating in a research recording, during which data from all available HD electrodes is captured. Inclusion criteria for patient seizure videos in this study were being >18 years old and having a spontaneous focal-onset seizure while undergoing HD recordings during a research task (e.g. passive listening, naming, language mapping). Ten patients between 2012 and 2021 met these focused criteria. Clinical data including sex (self-reported) and age were obtained from retrospective chart review (Table 1).

**Table 1:** Demographics and pertinent clinical information for the ten patients included in the scoring analysis.

ID	Sex	Age (y)	Onset age (y)	First condition shown	Depth	Subd.	Total electrodes	Implant laterality	SOZ laterality	SOZ localisation	Engel outcome
1	Female	30	28	LD	–	Yes	256	L	L	Posterior superior temporal gyrus	IA
2	Female	21	3	HD	Yes	Yes	362	L	L	Inferior lateral temporal region	IVB
3	Male	21	15	LD	Yes	Yes	366	L	L	Superior temporal gyrus	IIIA
4	Female	40	4	HD	Yes	–	120	B	L	Medial temporal lobe	*N/A*
5	Male	20	16	HD	–	Yes	322	R	R	Posterior margin of the superior and middle frontal gyri	IA
6	Male	24	18	LD	Yes	Yes	318	R	R	Inferior temporo-occipital junction	IA
7	Male	46	35	LD	Yes	Yes	342	R	R	Medial temporal lobe	IC
8	Female	40	1	LD	–	Yes	274	L	L	Frontal lobe and superior parietal lobe	*RNS*
9	Male	31	23	HD	Yes	Yes	212	L	L	Anterior medial basal temporal and medial occipital lobes	IA
10	Female	50	16	HD	Yes	–	84	B	L	Medial temporal lobe	*DBS*

The SOZ localisation is the original clinically determined seizure-onset zone determined from retrospective chart review. The number of original ICEEG electrodes in the recording (HD condition) is in the “Total electrodes” column. Abbreviations: ID, patient study identifier; y, year; Subd., subdural; HD, higher-density; LD, lower-density; R, right; L, left; B, bilateral; RNS, responsive neurostimulation; DBS, deep brain stimulation; N/A, not applicable (did not undergo resection nor neuromodulation treatment).

### Preprocessing and electrode density subsampling

The HD seizure recordings were referenced to a subgaleal electrode and sampled at 3052 Hz. These referential recordings were low-pass filtered to <255 Hz (anti-aliasing) before resampling to 512 Hz. Channels with poor signal were excluded, and notch filters at 60 Hz and harmonics were applied.

The referential LD recordings were created by subsampling HD recordings prior to rendering heatmap OPSCEA seizure videos. For grids (4 mm electrode spacing; HD), we digitally omitted every other row and column, resulting in 8 mm spaced LD recordings analogous to our clinical recordings described above. For depth probes (5 mm-spaced electrodes), we similarly omitted every other electrode to create 10 mm LD spacing.

### Heatmap visualisation

To minimise potential bias interpretation related to knowledge of the density condition, we incorporated approaches from the rapidly expanding field of quantitative EEG and data visualisation. OPSCEA^11^ is a quantitative ICEEG data review approach recently validated as a helpful adjunct for identifying the SOZ, with accuracy similar to the gold standard (clinical teams using traditional trace-based recordings), and even relation to treatment outcomes.^11^ Specifically, OPSCEA colourises a digitally reconstructed brain surface with a heatmap according to the intensity of the seizure activity on nearby electrodes. Seizure activity is estimated using a line-length transform metric that is normalised (z-scored) for each channel to a pre-ictal baseline. Heatmap colourisation of each location on the brain utilised values based on seizure intensity from nearby electrodes with a distance-based Gaussian drop-off function. It was adapted for this study from the additive approach in the original method (synergistic influences of electrodes in closer vicinity,^21^ which would be confounded due to density condition) to an adapted maximum-based approach in which colour data was assigned by taking the maximum from all electrodes in the vicinity.

Many SOZs are deep targets (e.g. hippocampus), hence the importance of SEEG and hybrid ICEEG recordings.^7^^,^^22^ As described in the original publication, OPSCEA provided additional brain views including all depth electrode recording sites via “omni-planar” slice views running parallel to each depth probe trajectory. The MRI voxels running along this plane illustrated the patient's individual anatomy (grey and white matter), upon which the heatmap visualisations were projected similar to the surface views, and scorers were able to annotate these slices for deep SOZ sites using the user interface in the same manner as cortical surface sites.

**Fig. 2:**
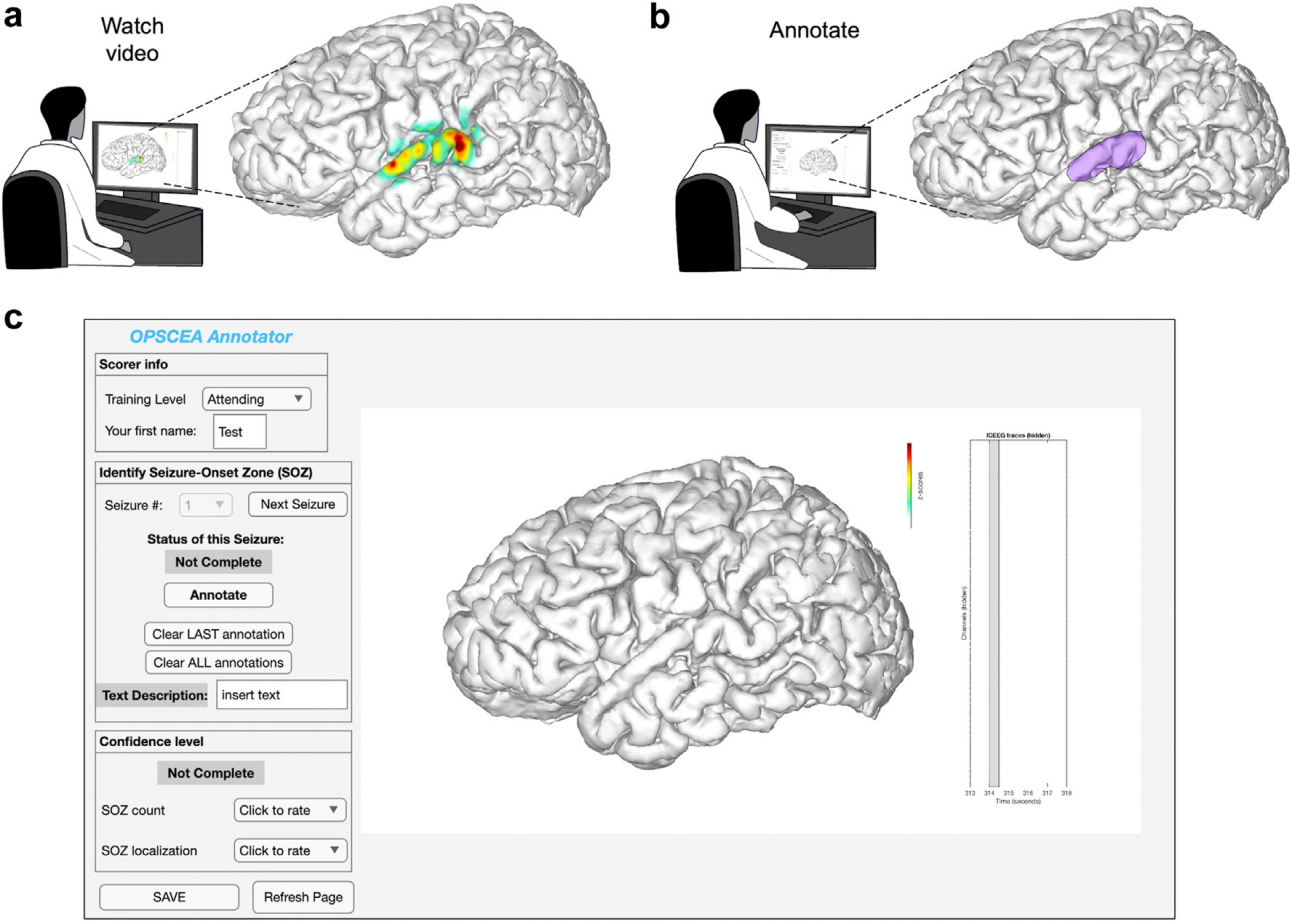
**Seizure viewing and annotation procedures. a.** Scorers watched individual seizure videos in blinded fashion (electrode dots hidden, no ICEEG traces; see Methods). Panel shows a heatmap of z-scored line length activity on the brain from an example video frame (seizure in Fig. 1c). **b.** Scorers subsequently annotated their SOZ area interpretation using a customised user interface (example from an individual scorer shown). **c**. User interface used for annotation and confidence levels (SOZ count-confidence level for the number of independent SOZ zones; SOZ localisation-confidence in the region annotated being the actual SOZ).

To enable blinding and to prevent bias to condition and direct comparison (HD, LD), we hid the electrode locations (Fig. 1b and c) from reconstruction heatmap videos. We also omitted raw ICEEG traces (previously displayed adjacently^11^), yet for temporal calibration we left the ICEEG axis intact to display time elapsed in seconds. A schematic for the scoring procedure is shown in Fig. 2a (watch video) and Fig. 2b (annotate), and Fig. 2c shows an example video screenshot displayed in the scoring interface for annotation. For each of the ten seizures, we made independent ictal heatmap (OPSCEA) videos for both HD and LD conditions (n = 20 videos).

### Ethics

This study was approved by the UCSF Institutional Review Board/Committee on Human Research (Study Number: 22-37089) and all patients and scorers provided written informed consent.

### Scoring participants and procedure

Participants were eligible if they were board-certified epileptologists at our institution (UCSF Medical Center) and willing to participate as scorers in the study by independently reviewing all seizure videos and using a computer interface to annotate them. For each OPSCEA video seizure, a screenshot still of the video (without heatmap) was displayed on a customised graphical user interface created in MATLAB (Fig. 2c), and the scorer annotated polygons outlining their opinion of the location of the likely SOZ (Fig. 2b). A five-point Likert-scale confidence rating was also given by scorers (1–5 for Uncertain, Possible, Likely, Probable, Certain).

A randomised single-blinded cross-over design was used, in which each scorer first scored each of ten seizures in OPSCEA, which were randomised for density by permuting the order of five instances of each density condition across the ten seizures (HD or LD, i.e., blocked randomisation by seizure; density order in Table 1; randomisation performed using MATLAB's *randperm.m* function completed by J.K.K.). Following a break in scoring, they then scored the same ten seizures in the opposite condition (LD or HD). Thus, the HD and LD conditions for a given seizure were separated by several other seizures in the interim as focused distraction. Importantly, scorers were not told they would see the same seizures more than once, and by extension they were thus blinded as to whether HD or LD was presented first for a given seizure (allocation concealment). Furthermore, scorers were not allowed to go back to view prior videos to compare nor to revise results, and they were not told the results of the study until all analyses were complete. A carryover effect was assessed between seizures in which the LD condition vs. the HD condition was shown first (both n = 5; see Table 1).^23^

### Statistics

All analyses were performed in MATLAB (version 2022b, Natick, MA, USA) including internal functions in the Statistics and Machine Learning Toolbox. For inter-rater reliability assessment, to help account for agreement that might occur by chance (e.g. SOZs from different patients have varying sizes in the annotated images) we utilised unweighted Cohen's kappa scores for a primary outcome. These scores were computed between each of the 15 unique pairs of 6 individual scorers (pair-wise scorer combinations) on the SOZ annotation region. Specifically, we evaluated whether the individual pixels highlighted by one scorer overlapped or not with pixels highlighted by the other scorer relative to all pixels in the image. We did this similarly for *intra*-rater assessment, using HD vs. LD conditions of the same seizure for each scorer. We based the retrospective clinically determined “correct” SOZ on the actual retrospective determination made by the clinical team using the available clinical pre-surgical workup information at the time of each individual patient case included herein. We evaluated whether the scorers included any or all of the SOZ anatomic area(s) (specified in the retrospective clinical team documentation) in their annotations for each patient, and if so this was deemed an “overlapping” annotation similar to our previous clinical validation study of the OPSCEA heatmap method.^11^

We used linear mixed effect models to study primary outcome measures of seizure onset zone area (spatial extent of annotated pixels, natural log transformed due to positive skew) and confidence level (numeric; see ordinal model below) in SOZ localisation. Independent variables were fixed effects of the density of electrode sampling (HD vs. LD; binary), whether the SOZ was classified by the scorer as multifocal or unifocal (binary), and whether the annotation overlapped with the correct clinical SOZ (binary), as well as random effects of scorer (six levels) and patient seizure (ten levels). Clinical variables (e.g. age, sex) were not built into the design of the study. In light of sample size limitations scorer and patient seizure random effects were modelled as uncorrelated. Given the relevance of inter-rater reliability relevance for scorer confidence,^24^ individual kappa scores (average of pairwise kappa scores with all other scorers) were also included in the confidence level model.

We assessed normality, homogeneity and sphericity,^25^ and linearity assumptions of our models using plots of model residuals. The dispersion of the residual data point clouds was checked for normality and homogeneity, along with the consistency and linearity of the variance across fitted values for our quantitative predictors. We also verified that our results using confidence level were robust to ordinal regression as an alternative model (cumulative logit link mixed model with Laplace approximation using the *clmm* function in R; Indianapolis, IN, USA).

The ten seizures, with six scorers and two conditions (HD, LD) for a total of 120 observations, were a retrospective convenience sample that leveraged a rare scenario. Nevertheless, we considered whether the available sample would be large enough to detect clinically important differences. For the primary HD vs. LD comparison, there were n = 60 vs. n = 60 observations, and we assumed a conservative design effect of 3.0 which would imply an effective sample size of 20 vs. 20 independent observations. This would give 80% power to detect a 0.9 SD difference between HD and LD scores, suggesting our study was likely powered to detect relatively large effect sizes.

### Role of funders

The funders had no role in study design, data collection or analyses, interpretation, or the writing of this manuscript.

## Results

**Fig. 3:**
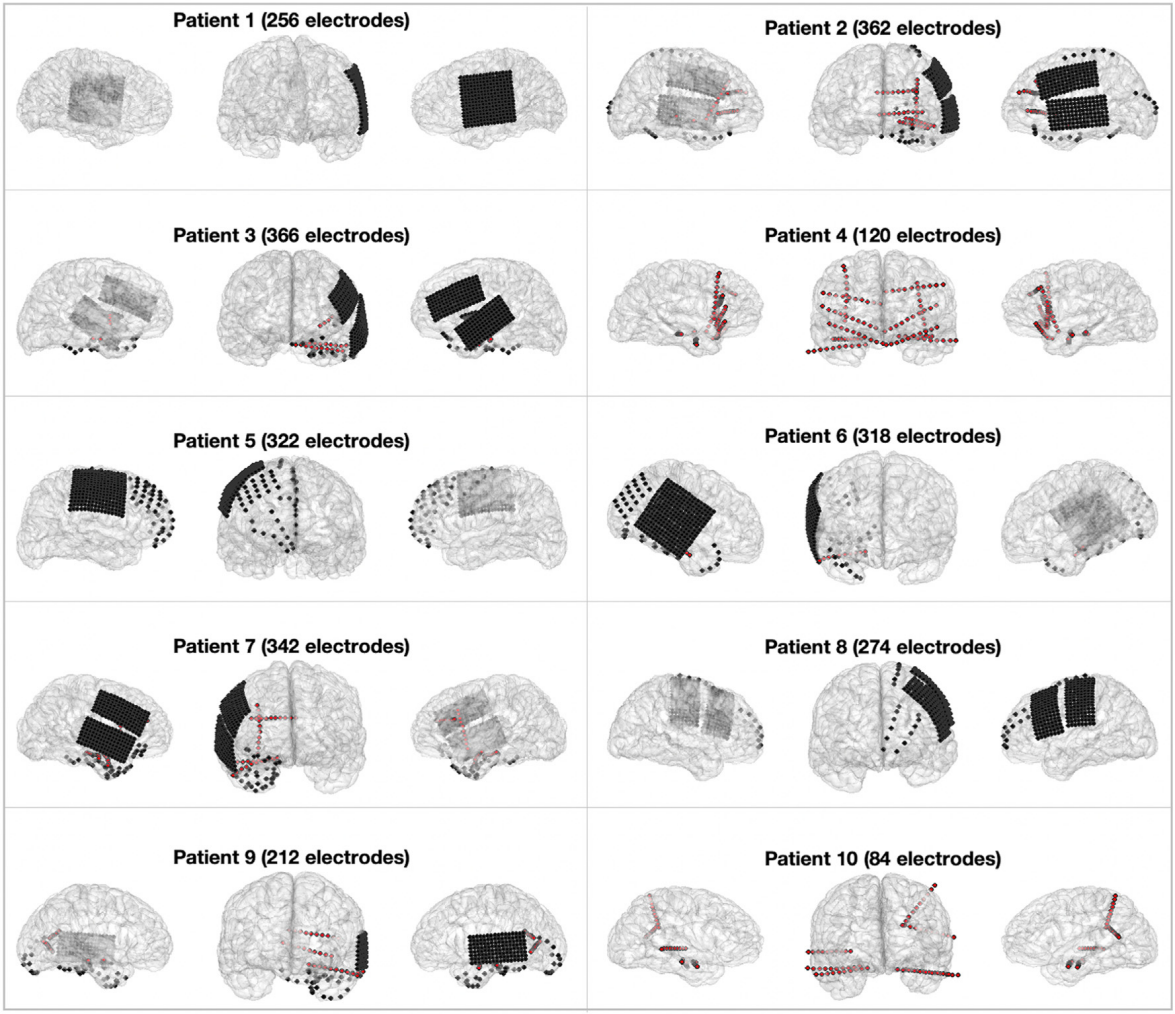
**Intracranial electrode coverage.** Semi-transparent views of each ICEEG patient's brain are shown as individual digital mesh reconstructions along with co-registered electrode locations (red dots indicate depth electrode contacts). Three panels are shown for each patient, providing different anatomical perspectives (panels from left to right: right lateral, anterior, left lateral).

There were 10 patients in the 2012–2021 study period who met the inclusion criteria defined in the Methods section. All recordings were of high quality and no patients were excluded. Clinical and demographic data is detailed in Table 1. Of the 10 patients 5 identified as female and 5 identified as male. Ages ranged from 20 to 50 years of age, and ages of epilepsy onset ranged from 1 to 35 years old (2–39 year history of epilepsy prior to invasive monitoring). The number of electrodes recorded ranged from 84 to 366 total per patient. There were 5 hybrid cases (depth probes and subdural grids and strips), 3 subdural only, and 2 SEEG (depths only). Among these, 5 were left-sided cases, 3 right-sided, and 2 bilateral (Fig. 3). SOZs were left-sided in 7 cases and right-sided in 3 cases (no bilateral multifocal cases). All patients had at least 2 years of post-operative follow up. Table 1 also shows the SOZ localisation and Engel outcomes for patients who underwent subsequent resection surgery.

### Annotation agreement

**Fig. 4:**
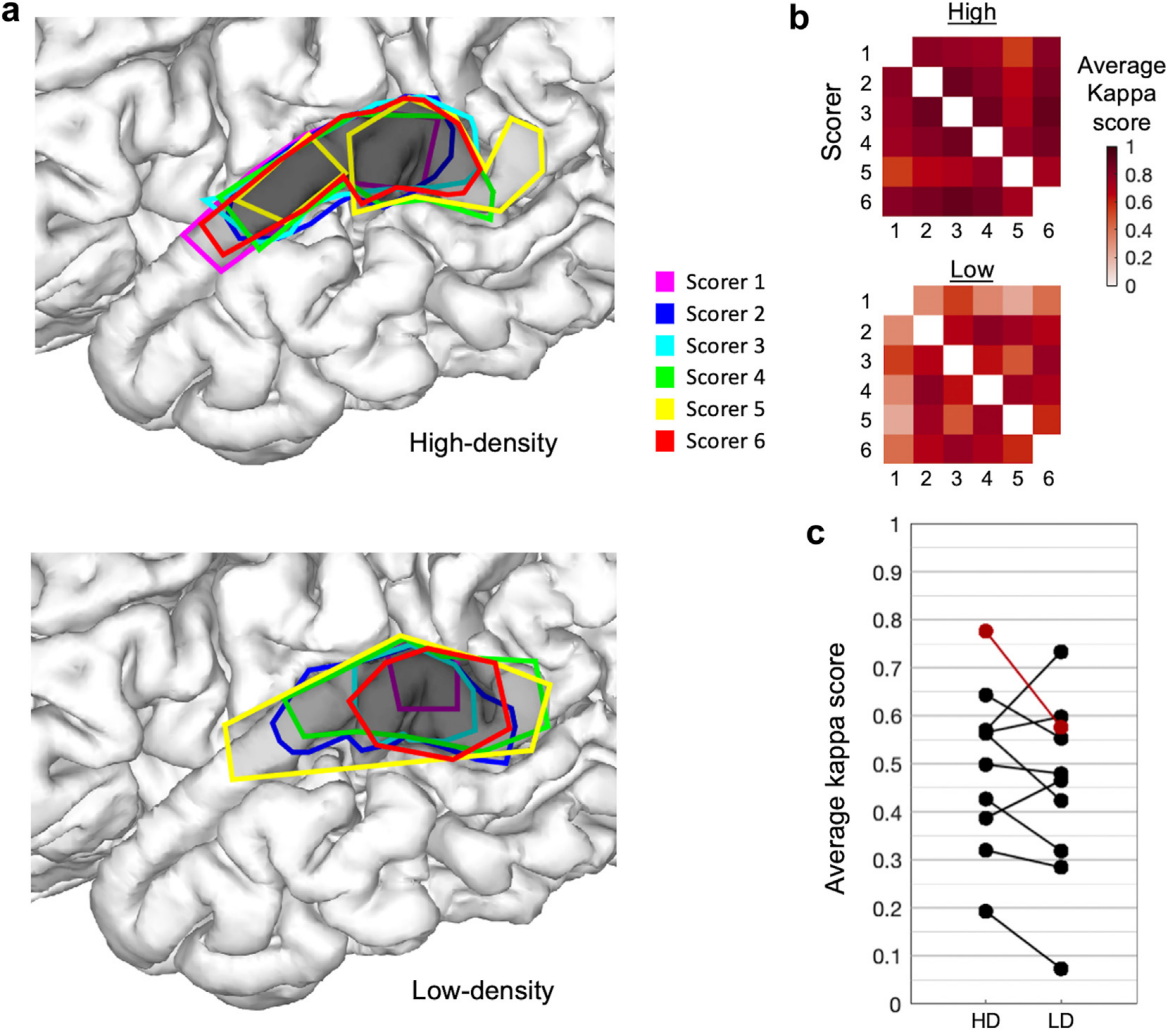
**Inter-rater agreement of SOZ. a.** The reconstruction view shown in OPSCEA videos for seizure 1 is shown with overlaid SOZ annotations for all scores (coloured outlines) for the HD (top) and LD (bottom) conditions. Darker shades of semi-transparent grey demonstrate overlap and, hence higher agreement across scorers. **b.** Pair-wise agreement (Cohen's kappa) between scorers for the HD (top) and LD (bottom) conditions for the seizure depicted in **a**. **c.** Kappa scores for all seizures (thick black lines; thick red line, for example, seizure in **a**, **b**) between HD and LD conditions averaged across all scorer pairs (n = 15 pairs averaged for each dot). Despite considerable variation across scorers (**a**, **b**) and seizures, the difference between HD and LD conditions is generally apparent as mostly negative line slopes for individual seizures.

Six board-certified epileptologists scorers agreed to perform the study and completed it with no missing data. Agreement between epileptologists on the extent of the SOZ (inter-rater reliability; Fig. 4a–c, pair-wise scorer combinations illustrated in Fig. 4b), assessed by overlap of the total area of annotations, was moderate overall across all 20 seizure videos (average unweighted Cohen's kappa: 0.47). Pairwise agreement for individual scorers (i.e., row averages in Fig. 4b for example seizure in 2a) across all seizures ranged from slight to substantial (0.07–0.78). Kappa scores were significantly higher for the HD condition (mean: 0.49 ± 0.17 standard deviation) than the LD condition (mean: 0.45 ± 0.19) and significantly different when adjusting for scorer and seizure (Coefficient: 0.044, 95% C.I. 0.007–0.081, p = 0.021, mixed effects model). Comparing the HD vs. LD conditions within each scorer (*intra*-rater reliability), we found a mean Cohen's kappa across all scorers and seizures of 0.56, with a range across individual scorers from 0.45 to 0.69.

### Seizure onset zone area

**Fig. 5:**
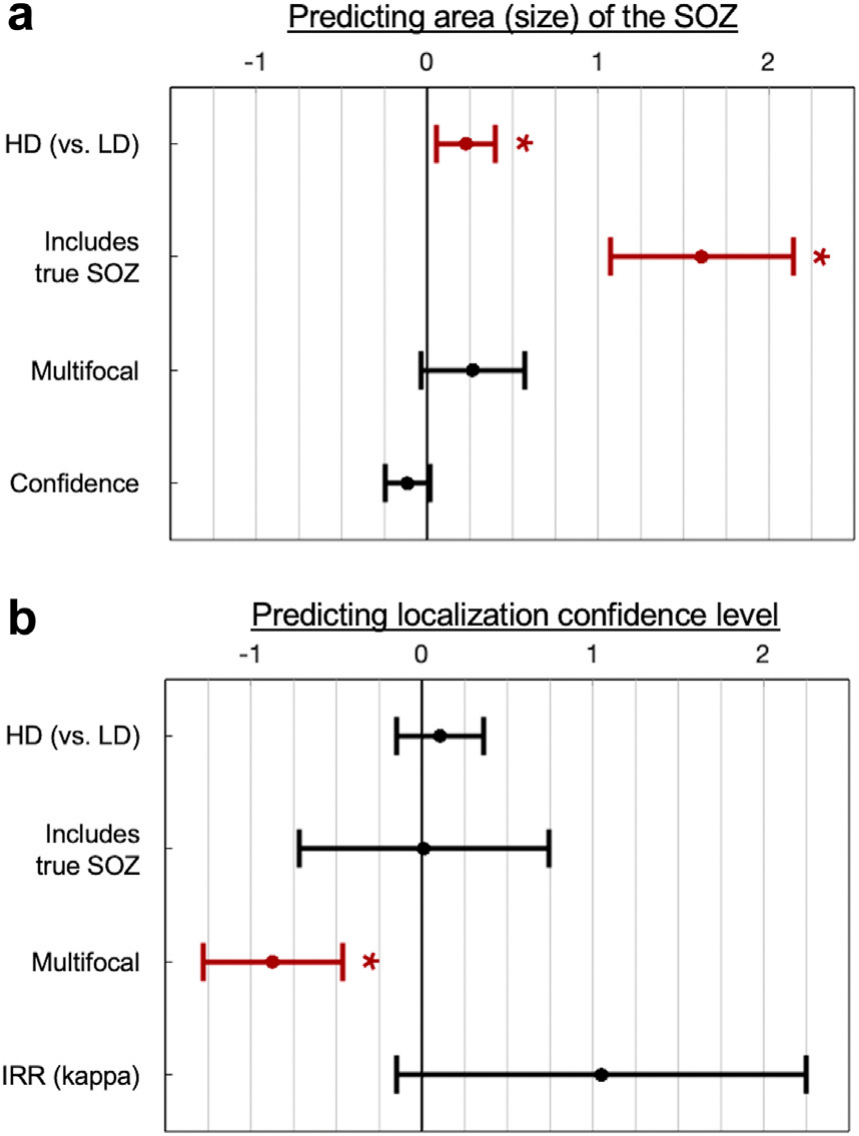
**Coefficient estimates for mixed effects models**. **a**. Coefficients and 95% confidence intervals for the mixed effect model that estimated area of the annotated SOZ (total number of pixels, natural log transformed). Predictors included whether the recording was HD (vs. LD) (p = 0.011), whether the annotation included the true clinically determined SOZ (vs. not) (p < 0.0001), whether the scorer qualified the SOZ as multifocal (vs. not) (p = 0.084), and level of confidence that the annotated SOZ reflected the true SOZ (vs. not; 5-point Likert scale with higher indicating more confidence) (p = 0.085). Predictors showing notable relations are shown in red with asterisks. **b.** Similar to **a** for the mixed effect model that estimated localisation confidence. Predictors included whether the recording was HD (vs. LD) (p = 0.410), whether the annotation included the true clinically determined SOZ (vs. not) (p = 0.978), whether the scorer qualified the SOZ as multifocal (vs. not) (p < 0.0001), and kappa scores (inter-rater reliability, IRR) (p = 0.086).

The spatial extent of the SOZ(s), measured as the total area of annotated image pixels, was significantly higher for the HD condition (i.e., lower for the LD condition; Coefficient: 0.227, 95% C.I. 0.052–0.402, p = 0.011, mixed effects model; Fig. 5a). In other words, on average HD presentations resulted in SOZ that were 25.4% times larger (e^0.227^) than LD presentations. Scorers’ annotations overlapped with the retrospective clinically determined SOZ (trace-based, based on chart review, see Methods) 91.7% of the time ( ± 3.9 standard deviation). A larger SOZ area was predicted by whether the annotation overlapped^11^ with the clinically-determined (trace-based) SOZ (Coefficient: 1.606, 95% C.I. 1.070–2.141, p < 0.0001), but not scorer confidence levels in localising the SOZ for a given seizure (p = 0.084) nor whether scorers designated the seizure as multifocal (vs. unifocal; p = 0.085).

### Scorer confidence

Scorer confidence in their localisation of the SOZ was lower if the SOZ was designated as multifocal (vs. unifocal; Coefficient: −0.873, C.I. −1.281 to −0.465, p < 0.0001, mixed effects model; Fig. 5b). There was no relation of scorer confidence to SOZ annotation agreement (kappa scores) across raters for a given seizure (p = 0.086), nor whether the annotation overlapped with the retrospective clinically determined SOZ (p = 0.978). Scorer confidence was not affected by electrode density, or in other words, whether the recording condition was HD (vs. LD; p = 0.410).

### Carryover assessment

In light of the crossover design of our study, we wished to assess whether the order of the density condition (i.e., whether LD or HD was delivered first for each consecutive seizure video) functioned as a carryover effect to influence our results.^23^ While this was unlikely with our design (balanced randomised density condition order for each consecutively viewed seizure; see Methods, Table 1), we fit alternative models in which the order of the density presented was added as a predictor. There was no effect of whether LD was shown first vs. HD shown first in the SOZ Area model (p = 0.652, mixed effects model), nor for the Scorer Confidence model (p = 0.431, mixed effects model), nor any notable changes in the significance of the fixed effects described above for these alternative models.

### SEEG simulation

**Fig. 6:**
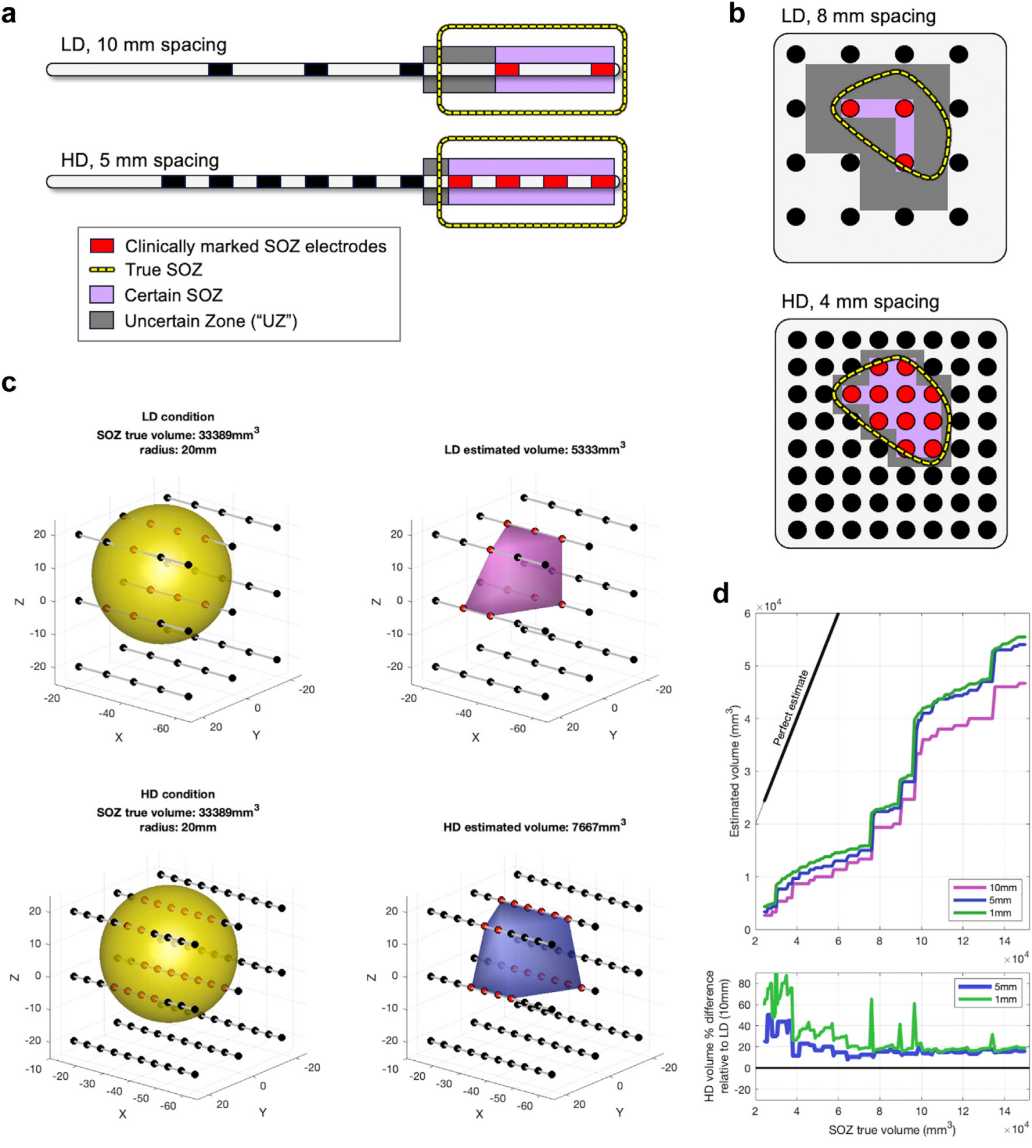
**Schematics and simulations of density influence on estimated SOZ size. a**. Linear electrode arrays (e.g. single depth probe or subdural strip) with HD (5 mm) and LD (10 mm) spacing are shown to illustrate the smaller observed SOZ (and thereby a larger uncertain zone, UZ) in LD due to the trade-off between sampled and unsampled tissue. Clinically marked SOZ electrodes are in red, unmarked in black. **b**. Two-dimensional electrode arrays (i.e., grids) with HD (4 mm) and LD (8 mm) spacing, similar to **a** in illustrating the smaller observed SOZ (and larger uncertain zone) in LD. **c**. Simulation of three-dimensional multiple-probe electrode arrays (i.e., SEEG) with LD (10 mm; top row) and HD (5 mm; bottom row) spacing. Similar to schematics in **a** and **b**, a theoretical “true” SOZ is simulated in yellow as a sphere, and the electrodes enveloped in this SOZ for each condition are annotated in red. These red electrodes are used to create a 3D “estimated” SOZ (i.e., the “Certain” SOZ from **a** and **b**). **d**. Estimated SOZ volumes are plotted as a function of increasing true SOZ volumes (spheres in left panels of **c**), compared to the ideal scenario (i.e., estimated volume = true volume; black line). Higher density sampling is always closer to the true SOZ size (top panel), and a percentage difference between the two (lower panel) demonstrates that SOZ size estimation with HD electrodes will always be larger than LD electrodes by roughly 5–50%.

The precise, true anatomical margins of the SOZ are unknown prior to resection (Fig. 6a–d), to both clinicians and researchers, hence the need for diagnostic testing including ICEEG for pre-resection approximation. We performed an SEEG simulation in which the true SOZ extent was known and scaled at different sizes, while the measured (observed) volume based on positive electrodes (electrode with SOZ boundaries) was compared between different density conditions. As demonstrated in Video 1 and Fig. 6c, the measured SOZ volume using HD (5 mm) spacing was consistently and 10–50% larger than when using LD (10 mm) spacing (aligning with the mixed model result of 25.4% above) and always closer to the true SOZ volume (Fig. 6d). Using very high density (1 mm) electrode spacing further improved estimation (i.e., closer to the true volume) beyond HD (5 mm) spacing.

## Discussion

In this study we used a single-blinded randomised crossover design to compare LD vs. HD resolution electrode sampling for clinical metrics of SOZ localisation. Trained epileptologists tended to annotate less tissue as involved in the SOZ and had less consistency with each other when viewing a LD recording compared to HD. Removing the SOZ more thoroughly is associated with a better surgical outcome, and therefore our findings of potential SOZ underestimation in LD recordings may be clinically problematic.^26^ In other words, we suspect recordings with higher electrode densities may help “move the needle” for the pervasive problem of surgically-refractory epilepsy, though further studies are imperative, as we were not sufficiently powered in this study to assess related outcomes.

This work adds further clinical motivation for recent advances in translational research using higher-density electrode arrays to capture epileptiform biomarkers, including focal interictal spikes and high-frequency oscillations with relevance for epilepsy surgery.^12^^,^^27^, ^28^, ^29^ While our study focused on the extent (3D margins) of the SOZ, it echoes concepts raised by previous work on the spatiotemporal scale of seizures. Highly focal seizures have been detected on micro-wires^30^ positioned between macrocontacts (e.g. microseizures), and detection at sub-centimetre scales is feasible through the addition of more electrodes (higher densities) on implanted arrays.^13^^,^^28^^,^^31^, ^32^, ^33^, ^34^ We would argue that depth or subdural arrays with either homogenous or hybrid micro-/meso-/macro-contacts may provide additional clinical benefit so long as the actual density of anatomical sampling is increased (Fig. 6) and factors such as electrode diameter and impedance are considered.^35^

Standard clinical ICEEG review (viewing voltage traces on screen) was not feasible for an unbiased evaluation of the influence of electrode density on seizure localisation because the sheer numbers of electrodes and their traces prevent blinding to condition (i.e., up to four-fold more traces on the screen for high density grids; Fig. 1a). Whether or how knowledge of electrode density might confound annotations was not clear prior to our results, but nevertheless, we strived to minimise theoretical risks of expectation bias (e.g. as consistently rating confidence higher (or lower) for the HD (or LD) condition). In addition to our single-blinded design (Methods), we utilised a recently described and validated method of direct visualisation of seizure activity (OPSCEA method) for this study in lieu of typical review of tracings. This data visualisation method enables anatomical approximation of the SOZ digitally *in situ* which relates to surgical outcomes in clinically-meaningful ways.^11^ Moreover, in this study it enabled direct visualisation of onset and propagation patterns yet with relatively blinded comparison of HD vs. LD conditions, and clinical relevance.^5^^,^^11^ Scorers were not told that they would see the same seizures in two different conditions, nor were they told to attend to the potential density as they annotated. Thus, while scorers may have been able to discern some level of electrode spacing in certain video heatmap appearances, it is unlikely that the results herein could be explained by this knowledge as a potential confound.

Adding more recording datapoints between existing electrodes enables the detection of problematic intermediate areas that would have otherwise been missed. Missing these intermediate extensions due to less electrodes (LD) would thus underestimate the SOZ further from its true extent (see simulation in Video 1, Fig. 6). Thus, the (roughly 25%) larger SOZs annotated in HD recordings may have been driven by the additional spatial detail, since additional electrode sites in the HD condition^36^ may reveal seizure activity that is present between, and sometimes even stronger than, adjacent LD electrodes (see arrows in Fig. 1b). These “uncertain zones” (UZ) are problematic because they obscure the extent and contiguity of SOZ tissue. A truly non-contiguous SOZ may also seem even less contiguous when fewer recording data points are used (see example in Fig. 1), presumably clouding the specific anatomic boundaries of the SOZ (i.e., where to draw annotation lines, and hence, resection margins). We speculate that this produced more variance between scorers, lowering inter-rater reliability. Furthermore, as with low resolution pictures or imaging studies, under-sampling can miss small margin extensions, irregularities, or contiguities in a lesion, leading to inaccurate lower-bound measurement of lesion area or volume (Fig. 6).

The issue of under-sampling (schematic in Fig. 6) is rooted in discretising a continuous space (brain anatomy) to a finite resolution (evenly spaced electrodes). It is related to the problems of resolution uncertainty and quantisation error in analogue-to-digital conversion. Despite being different modalities, ICEEG under-sampling has some similarity to the partial volume effect in biomedical imaging, which can be especially problematic for smaller regions of interest.^37^, ^38^, ^39^ In addition, due to relatively small electrode contact sizes, tissue located in gaps between electrodes is not sampled at all aside from volume conduction to nearby electrodes.^28^ Thus, compared to biomedical imaging in which voxel boundaries are adjacent, this problem may be more pervasive in ICEEG recordings.

The additional spatial detail afforded by HD recordings may be most relevant for low-voltage fast activity (LVFA), an electrophysiological signature which generally represents pathological activity directly under the electrode contact.^40^ Spiking and other epileptiform activity patterns of sufficient voltage amplitude are often seen on nearby intracranial electrodes due to volume conduction, though nearby dipoles can go undetected if oriented orthogonal to electrode position. More importantly, LVFA constitutes the most common seizure onset pattern at roughly 40–50% of intracranial recordings for drug-resistant epilepsy.^40^ These aspects suggest that our results may be relevant for the majority of intracranial recordings. The trade-off between the certain SOZ and the UZ (Fig. 6) is also applicable to the intraoperative environment and bedside functional stimulation mapping of eloquent cortex, or when evaluating for interictal activity including spikes and HFOs if using in consideration of resection margins.^12^^,^^27^^,^^41^

Importantly, SOZ margin precision is most relevant for tailored surgeries, but not necessarily surgeries with pre-defined margins, particularly standard anterior temporal lobectomies (ATLs). Caveats could include tailored ATLs such as when the SOZ is determined to have cortical extension beyond the standard ATL margins, or when more posterior extensions of deep targets (e.g. hippocampal tail) are involved.^42^ HD recordings may also be beneficial in situations where the SOZ abuts eloquent cortex for more precise functional margins defined by electrical stimulation mapping.^36^

The inter-rater agreement of anatomical SOZ annotation across epileptologists/scorers was moderate, a pervasive problem described previously in studies using ICEEG^40^^,^^43^^,^^44^ and scalp EEG.^45^^,^^46^ This ranged up to substantial agreement depending on the seizure/patient in this study (Fig. 3c). We adjusted for this variation across different scorers and seizures using mixed effect models, revealing higher agreement for HD recordings relative to LD. This HD-associated improvement in agreement has clinical implications considering how the pervasive issue of inter-rater reliability may undermine clinical seizure localisation.^40^^,^^43^^,^^44^ Prospectively testing whether higher electrode densities improve inter-rater reliability and whether this is associated surgery outcomes would be beneficial in future work.

Subjective confidence ratings for seizure localisation using a Likert scale provide a clinically relevant measure for individuals^11^ but these were not significantly associated with electrode density, nor inter-rater reliability for a given seizure. Instead, confidence was inversely related to SOZs being reported as multifocal (vs. unifocal). These latter results potentially reflect uncertainty subjectively for cases in which the SOZ was more obvious (thus more consistent across scorers) or had more complexity (multifocal networks).

The surgical margin extent and clinical confidence results of our study pertain to any intracranial electrode recordings (Fig. 6), i.e., both SEEG and subdural recordings, as demonstrated by our results and the simulation in Video 1. Most patients herein underwent recently-described “hybrid” recordings at our centre.^7^ These consist of both depths and grids simultaneously, thus sampling both deep and superficial targets and affording detailed 3D anatomical coverage. The annotated SOZs herein included deep targets captured on depths (e.g. medial temporal; Table 1). Our use of HD grids provided numerous additional data points with regularly spaced sampling in two dimensions (2D),^36^ which may translate more directly to the 2D annotation on image screenshots performed by the scorers and that observed by the surgeon in the operating room, though depth electrodes including in SEEG and hybrid recordings provide spatial information for reconstructing ictal spread and propagation patterns as well.^11^^,^^16^^,^^17^^,^^47^

The full 3D volume of the functional SOZ is always an estimation in both clinical and research contexts. It is impossible to precisely determine its putative true extent prior to resection, particularly for non-lesional cases. Corroborating our results, we demonstrate through simulation that higher electrode densities provide closer approximation to the true 3D volume of the SOZ in simulations, and this remains true with “very high” density sampling (1 mm; Video 1, Fig. 6c). However, a plateau effect is still evident, illustrated as the gaps between HD (5 mm) and higher (1 mm) density sampling compared to the true simulated volume in Video 1 and Fig. 6c. This remaining gap is related to the overall extent of coverage, or in other words the inter-component spacing (e.g. depth-to-depth, depth-to grid, etc.). While the number of implanted components is limited for clinical reasons (e.g. risks of haemorrhage, infection),^48^, ^49^, ^50^ we would argue that underestimation due to lower densities on the components that are *already implanted* is unnecessary. Our results suggest that higher densities on already-implanted components can help maximise their available diagnostic return (demonstrated in Fig. 6 and Video 1). Additional localisation techniques such as intracranial electrical source localisation are helpful to account for such gaps in coverage, and importantly, higher electrode densities (especially focused around the SOZ) are dually helpful for source localisation methods as well, and future studies to this effect may be helpful.^14^^,^^15^^,^^51^^,^^52^

Increasing the density of recordings requires attending to more channels on the screen, yet this issue has been recurrently overcome for scalp EEG and ICEEG ever since the transition from analogue (paper) EEG to digital in the past few decades. Both intracranial and high-density scalp recordings have required higher capacity amplifiers, and indeed, many systems now support hundreds of channels for clinical purposes.^53^^,^^54^ Seizure patterns remain easily apparent in the trace-based ICEEG of HD recordings (Fig. 1a, left panel) and contemporary computer monitors display such detail. Moreover, quantitative EEG and data visualisation methods to assist clinical interpretation are rapidly increasing for both scalp and intracranial data,^11^^,^^16^^,^^17^^,^^55^^,^^56^ and generating lower sampling if/when needed from higher density arrays is feasible and validated.^35^ The expanding clinical utility of such quantitative and computational tools in digital scalp and intracranial EEG is increasingly clear, and further studies comparing more such tools to traditional trace-based review are needed.

Limitations of this study include a relatively limited patient pool due to the rarity of capturing seizures during HD recordings during research participation (see Methods—Statistics section regarding sample size considerations). We were relatively underpowered to assess differences in surgical/Engel outcomes (only 7 patients underwent resection), but future studies with larger numbers of patients may better delineate whether the influences of recording density on SOZ localisation are also predictive of better outcomes. Our relatively large confidence interval estimates for certain factors may also suggest a sparse data bias, and the results should be taken in context with their coefficients and measures of variance/confidence intervals.^57^, ^58^, ^59^ However, because our data are balanced by rater, seizure, and HD vs. LD condition, we suspect that our statistical results are robust to method choice. Another limitation was the use of a quantitative heatmap metric compared to traditional trace-based review (though this would have compromised density condition blinding), and 2D annotation on images for 3D reconstructions (area instead of volume, and without factoring in other anatomical factors such as specific gyri, sulci, eloquent cortex, and white matter pathways). This may have limited the precision of SOZ estimates compared to traditional comprehensive review (though see simulation in Fig. 6c and Video 1). However, our crossover design–marking the same seizure in both HD and LD conditions in a blinded fashion and randomised order for each seizure–mitigates these factors. While 7 of 10 patients had depth electrodes (SEEG or hybrid approach, Table 1) and our results apply to any implanted hardware (Fig. 6, Video 1), future studies of electrode density focused on SEEG approaches (depths only) may be most prudent because the vast majority of surgical epilepsy centres have prioritised SEEG almost exclusively in recent years.^8^^,^^9^

SOZ localisation in ICEEG is highly dependent upon pre-implantation hypotheses and related optimisation of electrode components for strategic coverage. However, our study reveals that higher ICEEG electrode densities on these already-implanted electrode arrays improve crucial functional anatomic detail and inter-rater agreement of SOZ delineation by epileptologists. Spatial aspects of diagnostic accuracy have driven previous advances in biomedical imaging (e.g. slice thickness), and similar future scaling of intracranial electrode densities may convey significant clinical benefit.

## Contributors

E.O.C.-E. and J.K.K. conceived and designed the study, and handled project administration, data curation, and analysis software. E.T. and J.P.A. contributed conceptually to adapting seizure heatmap data visualisation software customised for this study. P.A.G., V.R.R., P.H., J.M.F., E.G.-G., M.H., E.F.C., and R.C.K. contributed to data acquisition. E.O.C.-E. and J.K.K. wrote the initial manuscript. S.C., J.P.A., P.A.G., V.R.R., P.H., J.M.F., E.G.-G., M.H., E.F.C., and R.C.K. provided significant review and editorial input for subsequent drafts. J.K.K., S.C., and E.O.C.-E. have accessed and verified the data and statistical analysis along with interpretation of results. All authors are accountable for the accuracy and integrity of the work and have read and approved the final manuscript to submit for publication.

## Data sharing statement

Open software is provided on GitHub for the OPSCEA method (https://github.com/Kleen-Lab/OPSCEA), and including the annotation user interface and simulation analysis described in this manuscript (https://github.com/Kleen-Lab/OPSCEAhilo). The de-identified participant level data are available upon publication of this article through reasonable request via email to the corresponding author.

## Declaration of interests

Grants or contracts non-relevant to the content herein nor the sources listed in the Funding section above include clinical trial contract 24-41330 from Neurona Therapeutics, Inc. (R.C.K.), and NIH grants 5R25NS070680-13 (J.P.A.), K23NS125123 (J.M.F.), R01NS114122-01A1 (sub-contract, M.H.), and R01MH122431 (P.H.). M.H. served as a consultant for Schlesinger Law Offices and J Supple Law, as well as served as an expert witness for Cook County Public Defender. Additional non-relevant funding sources are NIH grants R01NS136739, UG3NS130527, UH3NS123310, R61NS125568, R01MH122431, R01MH123770, U01DC018671, DP2MH126378, U01NS117765, UH3NS115631, R01DC012379, R01GRANT13824497, and New Venture Fund #011931-2020-07-01 (E.F.C.), as well as NSF CogNeuro grant 2148753 (J.K.K.). All other authors declare that they have no conflict of interest.

## Appendix A Supplementary data

VIDEO **SEEG electrode density simulation.** Left sided panels show a three-dimensional multiple-probe electrode array (i.e., SEEG, nine depth probe components in the left hemisphere; MNI average brain), with LD (10 mm; top row) and HD (5 mm; bottom row) spacing. A theoretical “true” SOZ is simulated in yellow as a sphere, and the electrodes enveloped in this SOZ for each condition are annotated in red. These red electrodes are used to create a 3D “estimated” SOZ based on those red electrodes only, for each condition (middle panels). In the right panels, the estimated SOZ volumes are plotted as a function of increasing true SOZ volumes (spheres in left panels), compared to the perfect scenario (i.e., estimated volume = true volume; black line). As illustrated, higher density sampling is always closer to the true SOZ size (top panel), and a percentage difference between the two (lower panel) demonstrates that SOZ size estimation with HD electrodes (blue line) was always larger than LD electrodes by roughly 5–50%. This difference was even more pronounced with higher density electrodes (1 mm spacing; green line).
